# Comprehensive Review on Bulla: Uses and Nutritional, Cultural, and Processing Perspectives of an Enset‐Derived Food in Ethiopia

**DOI:** 10.1155/ijfo/2250084

**Published:** 2026-08-13

**Authors:** Getu Weyya, Habtamu Admassu, Eneyew Tadesse, Hirpasa Teressa, Tariku Tagesse

**Affiliations:** ^1^ Department of Chemical Engineering, College of Engineering and Technology, Wolkite University, Wolkite, Ethiopia, wku.edu.et; ^2^ Food Process Engineering Program, Addis Ababa Science and Technology University, Addis Ababa, Ethiopia, aastu.edu.et; ^3^ Biotechnology and Bioprocess Centre of Excellence, Addis Ababa Science and Technology University, Addis Ababa, Ethiopia, aastu.edu.et; ^4^ Department of Biology, College of Natural and Computational Sciences, Wolkite University, Wolkite, Ethiopia, wku.edu.et; ^5^ Department of Economics, College of Business and Economics, Wolkite University, Wolkite, Ethiopia, wku.edu.et

**Keywords:** bulla, enset, *Ensete ventricosum*, functional properties, nutritional composition, traditional foods

## Abstract

Enset (*Ensete ventricosum*) is a perennial herbaceous monocot belonging to the Musaceae family. It is a crucial crop in Southern and Southwestern Ethiopia, providing food security for over 20 million people. The three main enset‐derived food products are kocho, amicho, and bulla. Most studies have focused primarily on kocho, with much less attention given to the characteristics, production methods, and uses of bulla. Despite being traditionally regarded as the highest quality enset product and its considerable importance, the existing literature on bulla production contains significant inconsistencies. Additionally, much of the current literature does not clearly distinguish between bulla and fermented bulla; these two terms are often used interchangeably, which creates confusion. This review synthesized existing knowledge on bulla processing, nutritional profiles, socioeconomic roles, and medicinal applications. Following PRISMA guidelines, 337 records were identified through database searching. After removing 98 duplicate records, 77 records from nonreputable sources, and 79 papers that lacked clarity or rational justification, 83 articles remained and were assessed for full‐text eligibility after rigorous screening. The result showed that bulla is primarily a product of mechanical juice extraction and sedimentation, not requiring fermentation for its primary form. It is an energy‐dense product with high carbohydrate but low protein content. Kocho gives less energy yield (6.5 MJ/kg) than bulla (8.5 MJ/kg). Fermented bulla is produced using traditional fermentation methods that vary among households and localities, and standardized fermentation protocols have not yet been well established. However, fermented bulla might exist as a secondary variant with a scarcity of standardized protocols for its processing. Previous in vitro and tablet formulation studies have suggested that bulla starch may have potential as a pharmaceutical binder and as a gelling agent in biotechnological applications. This review urges further empirical research to standardize processes, maximize production, and expand bulla′s applications for enhanced food security, medicinal uses, and economic benefit.

## 1. Introduction

Enset (order Zingiberales, family Musaceae, genus *Ensete*, and species *Ensete ventricosum* [Welw.] Cheesman) is a perennial, herbaceous, and monocotyledonous crop [1–3]. It is commonly known as the Ethiopian banana or false banana because it resembles the domesticated banana (*Musa* spp.). Like the banana, enset is a nonwoody, monocarpic, evergreen perennial and diploid plant with 18 chromosomes. However, unlike the banana, the underground corm and pseudostem are the edible parts of the enset [4]. *E. ventricosum* is one of the most important plant species in Ethiopia for its multipurpose use as human food, animal fodder, food packaging/wrapping material, traditional medicine, fiber for construction/rope, decoration, shading other crops, and source of income for many farmers. The plant reaches 5–7 m in height with developed pseudostem and corm [5, 6].

The genus *Ensete* has approximately seven to eight recognized species, whereas early classifications suggested a higher number. It is found throughout sub‐Saharan Africa (with three species), Madagascar (with one species), and southern Asia (with three species) [3, 6], having an ecological range from 1100 to more than 3000 m.a.s.l. [7]. In Africa, it grows commonly across the eastern, central, southern, and western regions of the continent [8]. Although the genus is widely distributed across Africa and Asia, enset (*E. ventricosum*) has only ever been domesticated as a food crop in Ethiopia, probably starting from 10,000 years ago [7]. Even within Ethiopia, enset mainly supplies sustainable food security for smaller southern and southwestern regions, where speakers of Semitic, Cushitic, and Omotic languages have inhabited [9–12].

Enset cultivation and production are particularly vital to the socioeconomic sustainability of communities in the south, southwestern, and central highlands of Ethiopia, where enset serves as a major staple food, source of income, livestock feed, and cultural resource [13]. The plant has the ability to withstand drought conditions [7], and over 20 million people utilize it as a staple food, making it a significant source of food [8]. From enset, mainly three different types of food products, namely, kocho, bulla, and amicho, are produced [14–17]. According to the Ethiopian Central Statistical Agency report, two million tons of enset products are harvested yearly [18]. The yields of kocho, amicho, and bulla were reported to be 27, 23, and 1 kg per individual enset, respectively [18]. This indicates that a large amount of enset food products is obtained from a single hectare of land, as 1000 to over 2500 individuals of enset can be cultivated on 1 hectare. Yield per hectare varies due to different reasons including heterogeneous agroecological conditions across enset‐cultivating regions, genetically distinct landraces, and age at harvest [8].

Bulla is the semiliquid component of enset harvested during the early stage of kocho processing [19]. Bulla is a starchy food obtained through the scraping and pulverization of enset pseudostem and corm tissues, followed by squeezing to extract the juice, which is then collected and allowed to sediment (decant). The resulting starch is subsequently dried (fine powder after drying) to produce bulla which is white in color [17, 18, 20]. This process does not involve fermentation, unlike kocho, although bulla is sometimes mistakenly considered a fermented food product [21].

Most studies have mainly looked at kocho [3, 22], whereas there has been much less focus on the characteristics, production methods, and uses of bulla [20]. Additionally, much of the current literatures do not clearly distinguish between fresh bulla and fermented bulla [11, 22–27]. The two terms are often used interchangeably, which creates confusion. Therefore, it is important to establish clear definitions, standard names, and proper production procedures. This will improve clarity, ensure product consistency, and facilitate the transition from traditional methods to industrial‐level processes. However, the traditional bulla processing method has several drawbacks such as hygiene issues, labor intensity, time consumption, high expenses, and yield loss [28]. Furthermore, food processing from enset is laborious and, therefore, requires technological innovation to simplify the process and enhance food quality [29].

This review is aimed at synthesizing the available literature on fermented and unfermented bulla extracted from enset. Specifically, it examines the processing methods, nutritional characteristics, and food applications of both products, while highlighting the distinctions between them. By bringing together the existing evidence, the review seeks to address inconsistencies and gaps in the literature and to identify areas requiring further investigation. The review is guided by the following research question: What is the current state of knowledge regarding the processing methods, nutritional composition, applications, and research gaps associated with unfermented and fermented bulla from enset?

## 2. Methodology

This review employed a systematic literature review approach to assess the existing body of published works. The methods followed a clear and logical process, involving the identification, evaluation, and analysis of published materials with a high level of rigor [30, 31]. To ensure transparency and quality, the guidelines used for systematic review [31] were followed during the search, selection, and review of the literature. The PRISMA flow diagram, incorporating the methods described in literatures [32, 33] for such reviews, was used to guide the review process, and the search covered up to at least 2025.

**Figure 1:**
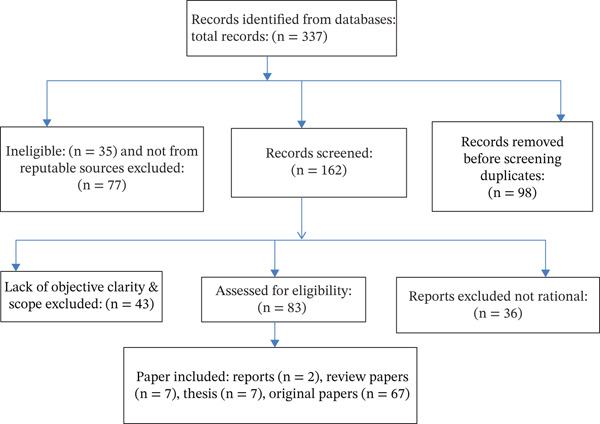
PRISMA flow diagram illustrating the identification and selection of research papers.

To gather a comprehensive dataset, the researchers conducted a keyword‐based search across major academic databases, such as Scopus, Web of Science, PubMed, and Google Scholar. Peer‐reviewed articles in English were included, and the search keywords were as follows: “enset,” “*E. ventricosum*,” “bulla,” “nutritional composition,” “physicochemical characteristics,” “proximate composition,” “fermentation,” and “functional properties.” Initially, 337 records were identified from the databases. After removing 98 duplicate records, 77 records from nonreputable sources, and 79 papers that lacked clarity or rational justification, 83 articles remained and were assessed for full‐text eligibility. As a result of this screening process, 67 original papers, 7 review papers, 7 theses, and 2 reports were deemed potentially relevant (Figure 1). Exclusion and inclusion of the materials were based on the levels of importance assigned to these items, with only papers meeting the high importance criteria being included.

The authors applied strict criteria to maintain the quality and relevance of the review by including peer‐reviewed articles, theses, and original research papers published in English in the paper. Conference abstracts and materials not related to enset products, mainly bulla, were excluded to ensure the quality of the review. These papers then underwent a screening procedure using a checklist that was assigned high or low priority to items such as title, abstract, introduction, methods, findings, and conclusion. A checklist consisting of six items, each with a high and medium level of importance, was used to select the papers for this review (Table 1). Eventually, the goal of this methodology was to reconcile significant inconsistencies in existing literature regarding bulla and fermented bulla and to establish a standardized understanding of its processing and multiple uses.

**Table 1:** Inclusion checklist of screening criteria for the papers.

Items	Criteria	Level of importance
Title	Classify the result as a systematic review, meta‐analysis, or both types of paper	High
Abstract	Clarify study details including background, objectives, methodological considerations, results, and conclusions	High
Introduction	Papers have a rational and objective approach	Medium
Methods	Data sources, sample size, collection, and analysis methods	High
Results	The tabular presentations, figures, and graphs	Medium
Conclusion	Conclusion of results and limitations	High

## 3. Result and Discussion

### 3.1. Overview of Enset

Enset is a resilient and versatile crop that has been domesticated in Ethiopia for its pseudostem and corm that are used to prepare various traditional foods [16, 29]. In addition to being drought‐resistant and providing high energy provision at low cost [34], it endures frost damage and withstands heavy rain and flooding [35]. Enset received the name the “tree against hunger” because of its exceptional role in ensuring food security during periods of drought, crop failure, and famine. Unlike annual crops, enset can be harvested at various stages of maturity throughout the year and maintained in the field as a living food reserve. Its contribution became particularly evident during the Ethiopian famine of 1888–1892 [7]. Enset‐based agriculture remains largely unfamiliar beyond its limited cultivation area, even though it grows wild throughout much of East and Southern Africa [8]. In Ethiopia, its cultivation has reportedly increased by 46% in two decades, whereas yield increased 12‐fold over the same period, making enset the second most produced crop species in Ethiopia [8].

Kocho is typically prepared from fermented pulp of pseudostem and corm of enset, whereas bulla is produced by dehydrating the juice extracted from the pulp of the pseudostem [15, 29, 36]. In comparison, kocho gives less energy yield (6.5 MJ/kg) than bulla (8.5 MJ/kg) even though it is rich in minerals. When compared with others, foods produced from enset contain higher energy that the body can metabolize than other crops cultivated in the country [37]. However, both bulla and kocho have less amount of protein [28] and fat [38, 39]. Amicho is another product that can be made from enset corm. Unlike bulla and kocho, corm contains a high concentration of calcium (Ca), Fe, Zn, K, and Mg [37].

Beyond its importance as a human food, enset products are used for different purposes. For instance, enset leaves and fibers are used in the packaging of bulla and kocho, whereas portion plates are used for pit lining that store kocho for microbial fermentation. The fiber is also used for making ropes, separating teff straw, and making sacks, bags, cordages, mats, and sieves [11, 40]. Sometimes, women′s skirt is made from fresh enset leaves and worn at ceremonies and markets [26]. Furthermore, the dried enset′s midribs and petioles are used as tying materials for house construction and firewood. They are also used for wrapping butter, pot stands, baby cushions, cleaning rags, and brushes [7]. Moreover, the plant is used for decoration, animal feed, and shading other crops like coffee, and its drought resistance makes it a risk‐avoidance crop [7].

#### 3.1.1. Harvesting and Preparation of Enset Pseudostem and Corm

**Figure 2:**
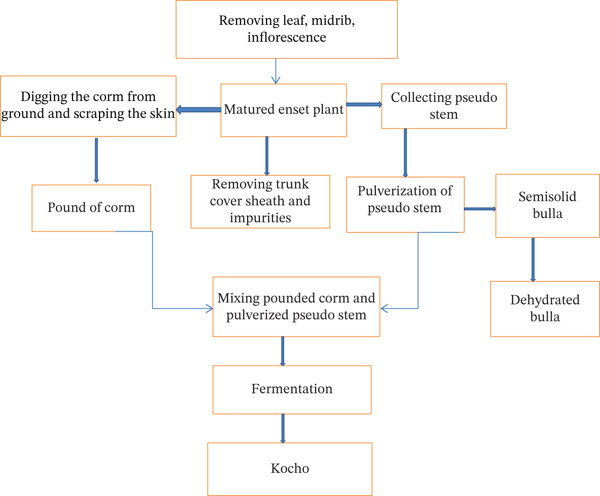
Traditional techniques for bulla and kocho production and processing.

Traditional processing of enset begins with the selection and harvesting of mature plants (Figure 2; techniques of bulla and kocho production and processing), typically between 3 and 7 years of age, based on large pseudostem girth and substantial corm development [8]. Mature plants are uprooted manually using machetes or farm knives, and the pseudostem and corm are removed from the field for further processing [9]. Once harvested, the outer layers of the pseudostem and the corm are decorticated to expose the internal fibrous and starchy core. Skilled processors, predominantly women, scrape the inner tissues with traditional tools such as bamboo splinters or metal scrapers to obtain a moist, fibrous pulp and facilitate subsequent fermentation and mixing with the pseudostem pulp [20]. At the same time, the corm is grated or chopped into small pieces to release starch.

#### 3.1.2. Nutritional Composition of Enset Parts (Pseudostem and Corm)

Enset dry matter comprises 90.87% organic matter and 9.13% ash. Organic matter is further composed of 74.57% soluble carbohydrates, 60.62% starch, 9.48% crude fiber, 5.98% crude protein, and 0.84% crude fat [41]. Different parts of enset have diverse contributions to the total dry weight. The pseudostem contributes the highest, whereas the leaf lamina is the lowest contributor [42]. Though unprocessed pseudostem and corm are low in protein content (4%), they are rich in other nutrients like soluble carbohydrates (80%) and starch (65%). Compared with other food items like potato, the corm possesses a significant number of amino acids [43]. In addition, most parts of enset are sources of important minerals such as phosphorus, potassium, magnesium, Ca, manganese, and iron [43]. Moreover, the details of nutritional contents of enset parts have been reported by several researchers as stated in Table 2.

**Table 2:** Nutritional analysis of enset pseudostem and corm.

Macronutrients	Unit	Pseudostem mean (SD)	Pseudostem min–max	Pseudostem samples	Corm mean (SD)	Corm min–max	Corm samples
Dry matter	% as fed	10.2 (4.8)	5.5–20.2	17	21.5 (4.6)	14.1–29.3	15
Crude protein	% DM	4 (1)	2.5–6.2	21	3.5 (1.1)	1.8–5.9	26
Crude fiber	% DM	12.3 (8.1)	5.8–23.2	5	9.3 (6.5)	3–17.5	5
NDF	% DM	56.6 (22.2)	17.6–84.2	18	46.4 (24.4)	13.1–89.3	15
ADF	% DM	14.5 (6.3)	7.4–27.5	18	6.9 (1.8)	4.8–11.3	15
Lignin	% DM	1.5 (0.7)	0.8–2.9	7	1.3 (0.7)	0.1–2.1	7
Ether extract	% DM	0.7 (0.5)	0.4–1.7	6	0.5 (0.2)	0.3–0.8	6
Ash	% DM	8.7 (2.1)	5.6–12.5	20	4.6 (1.1)	3–7.4	42
Starch	% DM	62.2 (7.5)	50–70	5	74.6 (6.6)	68–85	5
Total sugars	% DM	1.6 (0.9)	0.5–2.5	5	1.5 (0.8)	0.6–2.2	4
Calcium	g/kg DM	9.8 (9.6)	3–34	16	2.2 (1.7)	0.5–6.1	17
Phosphorus	g/kg DM	2.6 (2.4)	0.8–8.8	16	2.5 (2.4)	0.8–8.8	17
Potassium	g/kg DM	38.3 (10.3)	20–52	16	19.6 (7.1)	8.6–30.6	17
Sodium	g/kg DM	0.1 (0)	0–0.2	11	0.1 (0.1)	0–0.3	11
Magnesium	g/kg DM	3 (3.5)	0.6–10.5	16	2.1 (2)	0.6–6.3	17
Manganese	mg/kg DM	56 (13)	40–82	12	31 (10)	10–47	12
Zinc	mg/kg DM	14 (32)	1–116	12	130 (53)	62–226	12
Copper	mg/kg DM	4 (3)	1–12	12	5 (3)	2–12	12
Iron	mg/kg DM	158 (49)	103–227	10	86 (58)	34–237	10

*Note:* Data in the table have been taken from different literatures [41, 42, 44, 45].

Various studies indicated that enset parts have different nutritional compositions [37]. This nutritional variation among enset types may arise from genetic differences among cultivars (clones), as well as environmental conditions, agronomic practices, plant age, processing methods [38], frequency of transplanting, soil type, method of cultivation, and climatic conditions [10, 46]. Study [47] indicated that bulla from Gewada enset clone has higher content of iron, fiber, carbohydrate, fat, and energy than others. Similarly, bulla from Yanbule clone recorded higher Ca concentration and ash content, whereas bulla extracted from Zereta and Messina clones has higher protein content than the remaining [47]. In the same way, Kinnare and Astare clones have higher crude fat, fiber, carbohydrate, protein, total ash, and moisture contents [46]. On the other hand, these nutritional contents of enset can be affected by drought. Drought resulted in highly reduced crude protein, starch, ash content, and concentrations of elements such as P and K in the corm and pseudostem. However, levels of Ca, soluble sugars, and crude fiber content significantly increased in the enset corm and pseudostem that are subjected to drought. In this regard, enset plants in drought have shown two to four times the increase in soluble sugars [45].

Moreover, through the fermentation period, the composition of enset foods shows significant variation. A decrease in total ash (16%), carbohydrates (34%), protein (15%), starch (23%), soluble sugars (93%), and reducing sugars (84%) has been reported after 7 weeks of fermentation [48]. In contrast to this, the amounts of nonprotein nitrogen and free amino acids have increased by six‐ and 1.6‐fold, respectively. Organic acids like isovaleric acid, acetic acid, lactic acid, *n*‐butyric acid, and *n*‐valeric acid are found to be the most abundant at the final stage of fermentation [48]. Regardless of these nutritional quantifications of enset fractions (pseudostem and corm) across several landraces, studies that directly assess nutrient bioavailability in human diets remain limited. Research that integrates bioaccessibility and bioavailability of key micronutrients (e.g., iron, zinc, and Ca) under realistic dietary scenarios would clarify the nutritional contribution of products. There is also an opportunity to link landrace diversity with nutritional outcomes, which could guide cultivation and processing strategies tailored toward enhanced dietary benefits.

### 3.2. Processing of Bulla and Fermented Bulla

Bulla is one of the main enset products prepared from the squeezed liquid of scraped pulp [17]. In the process, after the starchy portion of the liquid is settled out, excess water left in the product is removed by evaporation or further squeezing to produce bulla, a white powder [17]. Later on, the product is rehydrated and used in making different dishes like porridge, dumplings, soup, and pancakes. In Southern Ethiopia, the main bulla‐producing regions, bulla is mixed with butter and spices to produce a dish like couscous. On the other hand, in Western Ethiopia, it is mixed with fresh milk and seasoned butter to produce a gelatinous food used in the preparation of different dishes [18].

Bulla production does not involve microbial fermentation. However, since traditional kocho processing often involves fermentation actions [4, 10], there is a widespread assumption that all enset products undergo fermentation. Such confusion among scholars might arise from the fact that kocho production involves fermentation with yeasts and lactic acid bacteria (LABs) which impart characteristics such as taste, texture, color, or aroma [48, 49], and bulla is also processed at the same time. However, kocho and bulla production follow two different processes, as shown in Figure 2. As indicated in the diagram, bulla is obtained at an early stage of kocho processing. During this process, bulla is separated from the long fibers of the scraped pseudostem using a split bamboo [20]. The product is then pressed; the liquid produced is collected and allowed to decant to produce bulla without any fermentation [20]. This is consistent with studies by some researchers [50–54], who reported that bulla is an unfermented enset product. Similarly, another study stated that bulla is a by‐product of kocho which is prepared from the liquid extracted when the scrapings and pulp of enset are squeezed [54] before kocho′s fermentation process begins. Bulla produced in this way can be used immediately or stored for later use. In addition, Fanta and Neela [55] diagrammatically showed that bulla production does not need a fermentation process.

On the other hand, bulla can also be produced through microbial fermentation (fermented bulla) [15, 24, 38, 48]. Fermented bulla refers to bulla that undergoes fermentation before or after drying [20]. In this process, after bulla is produced by the aforesaid method, the starch is left to ferment with microbes.

Researchers [20] carried out a laboratory investigation to check if there is a nutritional change in bulla after weeks of microbial fermentation. Accordingly, bulla was collected from four varieties of enset (Zereta, Yanbule, Gewada, and Messina: improved enset varieties collected from Areka Agricultural Research Center), and the samples were divided into three batches as soon as they were taken to Addis Ababa University, Food Science and Nutrition Laboratory. Food analysis was carried out for the first batch before fermentation, and the second and third batches were allowed for microbial fermentation for 15 and 30 days before nutritional and antinutritional analysis. The results showed that the major proximate compositions in bullas such as ash (in all four varieties), crude fat (in the Messina variety), carbohydrate (in Yanbule, Zereta, and Messina varieties), and energy kilocalorie in the Messina variety did not show significant differences when tested before fermentation and after fermentation, although the authors concluded it oppositely (Table 3). On the other hand, the fiber and moisture contents of bulla were significantly reduced as fermentation time increased. In addition, a few proximate compositions including crude protein showed an increase with the time of fermentation until 30 days. Fermentation also reduces the antinutritional components such as phytate and condensed tannins [34]. Increased phytase activity hydrolyzes phytate, leading to a reduction in phytate content and the release of bound minerals and proteins [35]. The decrease in tannin is nutritionally positive because high concentrations of tannins in the food inhibit the enzymatic activities of digestive enzymes, and this in turn interferes with the digestion of different food items. An increase in tannins could result in mucosal damage of the gut, making the substance undesirable for human consumption [37]. In contrast, [20, 48] reported that there were significant reductions in total carbohydrates (34%), total protein (15%), starch (23%), ash (16%), soluble sugars (93%), available carbohydrates (51%), and reducing sugars (84%) in enset food products after 7 weeks of fermentation. There was also a reduction in mineral concentration like phosphorus (29%), Ca (51%), and iron (15%), as fermentation time increased. However, the amount of organic acids such as isovaleric, acetic acid, lactic acid, *n*‐valeric acid, and *n*‐butyric acids and free amino acids increased with fermentation time [49].

**Table 3:** Fermentation effect on bulla′s proximate composition for four enset varieties [20].

Proximate composition	Varieties	Duration of fermentation (raw)	Duration of fermentation (15 days)	Duration of fermentation (30 days)
Moisture	Yanbule	52.5 ± 0.08^a^	51.1 ± 0.36^b^	49.87 ± 0.41^c^
	Gewada	54.1 ± 0.45^a^	50.97 ± 0.31^b^	50.27 ± 0.34^b^
	Zereta	49.57 ± 0.29^a^	48.38 ± 0.52^b^	47.88 ± 0.32^b^
	Messina	48.55 ± 0.11^a^	48.07 ± 0.17^a^	47.76 ± 0.91^a^

Crude protein	Yanbule	0.19 ± 0.003^c^	0.37 ± 0.005^b^	0.56 ± 0.01^a^
	Gewada	0.18 ± 0.005^c^	0.54 ± 0.01^b^	0.72 ± 0.016^a^
	Zereta	0.36 ± 0.005^c^	0.54 ± 0.013^b^	0.73 ± 0.005^a^
	Messina	0.36 ± 0.011^c^	0.58 ± 0.011^b^	0.91 ± 0.01^a^

Crude fiber	Yanbule	1.02 ± 0.01^a^	0.79 ± 0.029^b^	0.41 ± 0.006^c^
	Gewada	1.04 ± 0.005^a^	0.88 ± 0.03^b^	0.62 ± 0.012^c^
	Zereta	1.04 ± 0.005^a^	0.81 ± 0.012^b^	0.56 ± 0.056^c^
	Messina	1.04 ± 0.01^a^	0.85 ± 0.017^b^	0.42 ± 0.004^c^

Ash	Yanbule	1.05 ± 0.19^a^	1.24 ± 0.06^a^	1.27 ± 0.06^a^
	Gewada	0.77 ± 0.06^a^	0.78 ± 0.06^a^	0.88 ± 0.08^a^
	Zereta	0.91 ± 0.06^a^	0.92 ± 0.08^a^	1.14 ± 0.08^a^
	Messina	0.77 ± 0.1^a^	1.05 ± 0.25^a^	1.25 ± 0.04^a^

Crude fat	Yanbule	0.29 ± 0.027^b^	0.34 ± 0.026^ab^	0.42 ± 0.052^a^
	Gewada	0.3 ± 0.027^b^	0.35 ± 0.027^ab^	0.45 ± 0.026^a^
	Zereta	0.23 ± 0.026^b^	0.4 ± 0.026^a^	0.47 ± 0.05^a^
	Messina	0.29 ± 0.026^a^	0.39 ± 0.027^a^	0.39 ± 0.027^a^

CHO	Yanbule	97.45 ± 0.21^a^	97.25 ± 0.002^a^	97.34 ± 0.01^a^
	Gewada	97.71 ± 0.03^a^	97.46 ± 0.11^ab^	97.34 ± 0.03^b^
	Zereta	97.45 ± 0.1^a^	97.33 ± 0.03^a^	97.11 ± 0.18^a^
	Messina	97.54 ± 0.1^a^	97.13 ± 0.28^a^	97.03 ± 0.004^a^

Energy kcal	Yanbule	393.19 ± 0.6^b^	393.57 ± 0.27^b^	395.36 ± 0.48^a^
	Gewada	394.24 ± 0.37^b^	395.13 ± 0.23^ab^	396.23 ± 0.41^a^
	Zereta	393.35 ± 0.14^b^	395.05 ± 0.42^a^	395.58 ± 0.27^a^
	Messina	394.21 ± 0.22^a^	394.38 ± 0.94^a^	395.29 ± 0.3^a^

*Note:* Each cell shows mean ± SD, and means in the same row with different superscript letters show significant difference (*p* < 0.05).

In addition to making some nutritional variations, although inconsistent among scholars, fermentation changes the flavor, aroma, texture, acidity, and microbial profile. Changes in sensory properties due to fermentation make fermented bulla unacceptable (like kocho) outside the enset‐producing regions [56]. It also has a sour taste and bad smell compared with the unfermented one. Moreover, in all studies done on bulla production and processing, we have not identified the addition of any fermentation starter like gemma or gamancho (mixture of several other plant species) as in kocho production [17, 56]. Researchers have not provided any technique for the fermentation process of bulla. On the other hand, it is locally known that traditional bulla producers allow the bulla to stay for weeks after production for storage purposes [57] until use and selling, or for thickening [58], but not for the fermentation process. During this stay, the semifluid juice of the product will dry up, and naturally occurring microorganisms in the product might ferment the food to some extent, and this could mislead researchers to say bulla is a fermented product. However, the fermentation process during storage does not add significant nutritional value to the product, as discussed earlier. Likewise, preliminary results of our observations of some of the bulla‐producing sites in Gurage and West Shewa zones confirmed that bulla production and processing do not involve any microbial fermentation. In our observations, we also came to know that bulla has a better odor and taste than fermented bulla. Such characteristics might make bulla be consumed throughout the country including outside the bulla‐producing zones even though the product is far less than the demand.

### 3.3. Regional Bulla Processing Variations and Cultural Techniques

In Ethiopia, there are many methods of bulla processing (Table 4). For example, in Hadiya Zone, bulla is prepared by squeezing starchy liquid from pulverized corms and scraped pseudostems [14], whereas in Gedeo Zone, it is extracted from grated corms and scraping leaf sheaths [58]. However, in Kembata Zone and Cheha Woreda of Gurage Zone, bulla processing involves spreading leaf sheath pulp and grated corm on enset leaves, mixing them, and squeezing to extract the starchy liquid [61]. Furthermore, among the Gurage community, bulla is also produced by adding water to decorticated pseudostem mass, decanting the pressed and settled juice, and filtering it through sieves and hands [48]. Moreover, in Sebat‐Bet Gurage, bulla extraction involves hand squeezing the mixture of water and chopped enset [59].

**Table 4:** Traditional extraction methods of bulla from enset across various Ethiopian regions.

Region, zone	Method of extraction	Reference
Gedeo Zone, Southern Ethiopia	Starchy juice is squeezed out from decorticated pseudostem and corm. It is pressed by hand, collected, and allowed to stand 1–3 days or weeks until thickened. Starchy liquid is extracted from decorticated pseudostem and corm or from fully fermented kocho. The thick liquid is allowed to dry, and this gives a white powder, bulla	[58]
Gedeo Zone, Southern Ethiopia	After pressing a freshly decorticated mass or fermented enset, the extract is collected and settles into a white powder, bulla	[48]
West Shewa Zone, Oromia	The decorticated pseudostem mass is squeezed using feet into a perforated sack, and the liquid is collected using a plastic screen. Then, the filtrate is allowed to settle	[48, 49]
Cheha District, Gurage Zone, Central Ethiopia	After the fresh pseudostem has been decorticated and manually pressed, the liquid is passed through a sieve and allowed to settle before being decanted	[22, 48]
Sebat‐Bet Gurage, Central Ethiopia	Water is added to the decorticated leaves and pseudostems, and then it is squeezed by hand to produce bulla	[59]
Southern and Southeastern Ethiopia	Bulla is separated from decorticated and chopped enset by adding water to help remove the bulla by using a squeezer. After that, the bulla is gathered in the receiving tank	[41]
Kembata‐Tembaro Zone, Central Ethiopia	Bulla is obtained after chopping pseudostem and corm, and then squeezing and decanting follow	[60]
Kembata‐Tembaro Zone, Central Ethiopia	Once the corm has been grated and the enset leaf sheaths have been decorticated, the two are combined and put in a pit lined with fresh enset leaves for 7–15 days. After fermentation, the mixture is removed, and the liquid starch is extracted by squeezing it. After settling, the starch is left to dry and turn into bulla	[61]
Hadiya Zone, Central Ethiopia	The starchy liquid is extracted from scraped pseudostem and pounded corm by squeezing. The resultant starch is concentrated into a white powder called bulla	[14]
Some zones of the southern region	The thick liquid from grated corms and scraped leaf sheaths is allowed to dry to produce bulla	[62]

### 3.4. Nutritional Composition of Bulla

Different studies identified that bulla comprises several food composites and minerals [15, 21, 63], as stated in Tables 5 and 6. Carbohydrate contents of bulla from different localities and landraces range from 66.01% to 97.71%, whereas fat contents range from 0.11% to 4.25%. Minerals like Ca, Mg, Fe, and Zn range from 59.25 to 416, 7.78 to 74, 1.50 to 48.2, and 0.16 to 19.2 *μ*g/g, respectively. Studies [28, 64] also identified similar mineral concentrations in bulla. In addition, a study [21] reported similar concentrations of Ca (168.15–317.98 mg/g), Mg (31.51–55.86 mg/g), and Zn (0.76–2.34 mg/g), but higher Fe (1.5–2.54 mg/g) and major proximate for four different raw varieties of enset. This shows that bulla is rich in the major food components and important minerals [15]; therefore, it is recommended to use it as a staple food for all age groups, at different meals including on special occasions, and to honor guests with protein supplementation. Despite existing studies on the proximate composition, mineral content, and some functional parameters of bulla from the pseudostem and corm of enset, structural properties such as rheological behavior, pasting profiles, thermal transitions, starch granule morphology and size, crystalline structure, and functional chemical groups have not been investigated. Without these critical characterizations, the potential use of bulla as a functional ingredient in food formulations and other applications cannot be fully understood or optimized.

**Table 5:** Proximate composition of bulla reported in different studies.

Parameter	Bulla from three different locations [63]	Bulla from four varieties [20]	Bulla from eight enset landraces [15]
Moisture (%)	7.31–21.46	48.55–54.10	48.45–50.55
Carbohydrate (%)	66.01–89.98	97.45–97.71	94.79–97.62
Protein (%)	0.23–1.06	0.18–0.36	0.63–1.94
Fat (%)	3.82–4.25	0.23–0.30	0.11–0.63
Fiber (%)	1.39–2.15	1.02–1.04	0.46–1.03
Ash (%)	0.30–13.47	ND	1.10–2.21
Energy (kcal/100 g)	ND	393.19–394.24	387.90–395.59

**Table 6:** Mineral composition of bulla reported in different studies.

Mineral	Bulla from Woliso and Welkite (mg/g) [26]	Bulla from four varieties (mg/g) [21]	Bulla from three different locations (mg/100 g) [63]	Bulla from eight enset landraces (mg/100 g) [15]
Calcium (Ca)	0.416	168.15–317.98	87.53–344.63	59.25–76.46
Magnesium	0.074	31.51–55.86	Not determined	7.78–17.15
Iron (Fe)	0.0482	1.50–2.54	3.75–17.66	3.62–5.52
Zinc (Zn)	0.0192	0.76–2.34	0.16–9.98	0.31–0.67

### 3.5. Application of Bulla

#### 3.5.1. Culinary Application of Bulla

Bulla is composed of over 90% starch on a dry weight basis [15]. It is used to formulate food products such as sauces, dressings, and bakery products due to its water‐absorbing capacity and gel‐forming abilities, making it a versatile ingredient with numerous functional properties. In addition, it is used in snack foods by blending 57.8% bulla, 18.9% pumpkin, and 23.3% amaranth flour [51] that increases its nutritional value. Rehydrated bulla can be used in various ways, including making pancakes and porridge [21, 65]. Bulla is also used in snack foods and traditional dishes across Ethiopia [21, 51]. In the southern regions, bulla is mixed with seasoned butter and spices to produce small grains much like couscous [17]. In Western Oromia, it is mixed with seasoned butter and fresh milk to produce a gelatinous food, dumplings, and soup preparation [8]. Bulla foods are extremely popular at restaurants that serve the Ethiopian delicacy Kitfo (raw minced beef mixed with butter and spice) [43].

#### 3.5.2. Uses of Bulla for Nonfood Applications

Beyond its culinary uses, previous research highlights the significant potential of enset starch in nonfood applications, particularly in the pharmaceutical and biotechnological fields. It has shown promise as an excipient in tablet formulations, acting as both a binder and a disintegrant [66].

##### 3.5.2.1. Pharmaceutical Application of Bulla

Enset starch is used in pharmaceutical applications as a tablet binder and disintegrant [17]. The binding and disintegrant properties of enset starch have been evaluated in paracetamol tablet formulations and compared with tablets prepared with potato starch [6]. The results revealed that enset starch had superior binding ability, producing tablets of lower porosity and friability, but higher crushing strength than potato starch [6]. Similarly, the binding and disintegrant properties of pregelatinized enset starch have been investigated [48]. The study found that pregelatinized enset starch demonstrated binding and disintegrant properties comparable to those of commercially available partially pregelatinized corn starch [11]. The use of starch as a pharmaceutical gelling agent has been reported, but is discouraged mainly because of the need for a high concentration and heating to obtain a viscous gel, the opacity of the formed gel, and its poor stability compared with other gelling agents [11].

##### 3.5.2.2. Application of Bulla in Biotechnological Fields

Bulla is a potential gelling agent in different laboratory applications [14]. For example, cassava can be micropropagated using bulla as a gelling agent at a concentration of 80 g/L, which reduces costs by 86%. It is also stated that in vitro germinated seedlings of the enset genotype Oniya can be cultured on gelled bulla [14]. In vitro propagation of Anchote, bulla is used as a gelling agent [67]. In addition to gelling, bulla has thickening and stabilizing properties [68]. Furthermore, studies indicate that bulla can serve as a cost‐effective alternative to conventional hydrocolloids like agar, xanthan gum, and carrageenan, making it an attractive choice for food manufacturers seeking to reduce production costs [69]. Starchy foods can function as thickeners, stabilizers, and gelling agents [70]. However, there is a significant research gap concerning its potential as a natural food additive. However, bulla′s particular applications as a thickener and stabilizer agent in modern food formulations remain underexplored.

### 3.6. Market Dynamics and Economic Value of Bulla

Enset cultivation and production are vital to the socioeconomic sustainability of communities in Southern Ethiopia [64], and about 20 million people utilize it as a staple crop, making it a significant source of food [14]. Due to its high quality as a result of concentrated starch content, good odor, palatability, health benefits, and freshness when compared with kocho, bulla is preferred and typically fetches higher prices per unit weight than other enset products in the local market [18, 38].

Value chain analyses also indicate that the marketing of bulla influences household food security and income levels: Participation in bulla marketing increases average annual incomes and enhances calorie consumption, suggesting that economic activity linked to bulla can be an avenue for improving livelihood outcomes [71]. Bulla occupies an important niche within the informal agrarian market system in Ethiopia. Market surveys have documented price differentials where bulla is sold at approximately double the price of kocho, reflecting its demand in urban and periurban markets. Bulla is white in color and relatively small in production quantity as compared with kocho, but it still fetches a much higher price [20]. However, it is collected and sold via informal channels with no formal export or national standard procedures, even though national demand for bulla can exceed local production volumes, highlighting a potential gap between supply and market needs.

Although bulla is a high‐quality enset food product with higher prices, many farmers prioritize kocho production because it is the principal staple food in enset‐growing communities and plays a critical role in household food security [8, 13]. In addition to its smaller quantity when compared with kocho [18], bulla extraction from the scraped pseudostem and corm may influence the composition and fermentation characteristics of the remaining material used for kocho production. Consequently, processing practices and the extent of bulla extraction can affect the quality, texture, and overall desirability of the resulting fermented kocho [17]. Despite its economic value, bulla is also primarily traded through informal market channels, with limited standardization and no formal export system.

### 3.7. Health Benefits of Foods Derived From Enset

Several ethnic groups in Ethiopia use various parts of enset to treat cattle and human illnesses [2, 72]. The most commonly used parts of enset for medicinal purposes are the leaves and the stem [73]. Amicho is also used to treat different ailments of humans and livestock [10, 74]. Various processed forms of enset foods provide many health benefits. Enset products are applied for purposes such as the expulsion of placenta, healing of bone fractures, and antimicrobial activity against viral, bacterial, fungal, and nematodal diseases in humans [2, 28]. According to reports, some enset types are used for healing, prevention, and other therapeutic uses [74]. Its high Ca, phosphorus, zinc, and potassium contents might be the reason for this health benefit [2, 37, 72].

A study showed that enset‐based foods have a low glycemic index, which can be beneficial for those with diabetes or at risk of developing it [75, 76]. In addition to lowering cholesterol levels [55], bulla can particularly improve gut health [17, 43] due to its richness in fiber, which is associated with good digestive health [55]. Studies have reported that dietary fiber controls risk factors like hypercholesterolemia, obesity, hypertension, Type II diabetes, and coronary heart diseases [77]. By lowering the absorption of carbohydrates in the digestive system, they prevent hypertension as well as obesity. Furthermore, it is suggested that dietary fiber plays a protective role against rectal carcinoma through increasing viscosity, fecal bulking, and contact time that reduce the production of anticarcinogenic small‐chain fatty acids within the gut and increase steroid deconjugation that further facilitates binding to carcinogens [77]. Research also identified that boiled corm/amicho of different enset varieties is used to treat broken bones, joint displacement, diarrhea, cirrhosis, and back pain when often consumed with milk [11, 58]. Moreover, phenylphenalenones, the unique compound identified from different enset varieties, have been proven to have anticancer, antibacterial, and nematicidal properties [11].

Enset like Astara is believed to have medicinal values and is used to treat humans and livestock [10]. When it is consumed with cheese, butter, and milk, its corm is used to stimulate placental discharge in both humans and cattle and reduces delivery‐associated stomachaches, but rarely induces abortion. Woman in Bonga is fed amicho of the Choro variety with butter and milk immediately after childbirth to stimulate placental discharge [74], whereas Asikala is administered in Sidama for the same purpose [78]. Amicho of both Asikala and Choro clones is also given to dairy cows to treat similar problems [74]. Likewise, amicho of Qibnar and Astara clones has the same medicinal value among Gurage communities as [73]. In addition, Kaffa farmers also culturally plant this clone in their garden to deter the evil spirit. The boiled corm of the Ariko clone is used in the treatment of people caught by the flu. Furthermore, some clones are used to reduce fertility in both humans and cattle [74]. To cure bone fractures and pus‐filled swellings, milk is combined with bulla of the Tayo clones in Bonga [74] and Genticha, Ado, Midasho, Kiticho, and Gediwocho clones in the Sidama Zone [78].

Therefore, across diverse Ethiopian ethnic groups, enset is broadly considered an essential medicinal plant. In this regard, reports indicate remarkable consistency in the phenotypic traits that characterize its medicinal value across different enset‐cultivating communities. As indicated in Shank and Ertiro [28], enset mostly used for medicinal purposes has reddish‐purple leaf blades with midribs and pseudostems.

Enset food products are highly starchy and energy‐dense yet relatively low in certain essential nutrients, particularly protein, some vitamins, and micronutrients [66, 79]. For example, bulla contains very low protein concentrations, like kocho, relative to more nutrient‐dense foods, highlighting its deficiency in protein and certain micronutrients that are crucial for a balanced diet [38, 66]. Moreover, promoting dietary diversity through agricultural diversification and food fortification can help rural populations move toward more nutritionally complete diets while maintaining traditional food practices such as enset [65].

### 3.8. Cultural Significance of Enset‐Derived Foods

Enset‐based foods are a significant part of the Ethiopian diet and provide great cultural and economic significance for several ethnic groups in the southern and southwestern regions [12, 78]. Enset is considered not only a food crop but also an important aspect of their cultural heritage. For instance, it has cultural significance in ceremonial events like weddings and funerals. Its leaves are used on tables for serving food during weddings; people beat pseudostems laid on the ground, and they put the leaves on their hands to connect them to enset. When the head of the household dies, the members of the clan cut enset plants to express their condolence and sorrow [25]. Particularly, Kaffa farmers cultivate different varieties of ensets in relation to myths, poems, rituals, and beliefs [74].

Another study also identified songs and rituals associated with enset in the Sidama culture [78]. The Gurage people, who are highly dependent on enset for their livelihood, are culturally tied to the plant and label themselves the “people of enset” [28]. The study further explained the essential role of the plant in the social and economic life of the Gurage communities alongside other crops such as khat and coffee [80].

### 3.9. Gender Roles and Traditional Labor in Enset Processing

Enset cultivation and processing are highly gendered activities within Ethiopian agrarian societies. Multiple studies describe enset as a “women′s crop” because women perform the bulk of labor‐intensive tasks such as scraping, pressing, fermentation, and preparation of enset products [81]. These tasks are time‐consuming and physically demanding, yet largely unpaid and undervalued within household economies.

Whereas men may participate in planting and land preparation, women dominate the processing stages that transform raw enset into edible products like bulla and kocho. This division of labor influences both the distribution of work burdens and access to economic benefits: Men tend to control the monetary returns from enset product sales and market interactions despite women contributing most of the labor. Gendered household dynamics further shape market participation. In some communities, women are the ones who choose to sell small quantities of processed enset products to meet routine household needs [82, 83]. Yet cultural norms and constraints limit women′s autonomy in negotiating market prices and accessing broader income opportunities.

### 3.10. Processing Challenges and Innovations

Processing enset into edible products such as bulla, kocho, and amicho remains highly labor‐intensive, time‐consuming, and physically demanding, posing major constraints for households that depend on these foods [31]. Traditional methods involve manual scraping, pulverizing, pressing, fermenting, and drying of the pseudostem and corm, often requiring hours of work per plant and typically undertaken by women. Surveys in enset‐growing regions of Ethiopia consistently documented that traditional enset‐processing tools are cumbersome, result in low productivity and yield losses, and involve unhygienic conditions that may affect product quality. Recent engineering work such as the development of an integrated enset‐processing machine combines multiple processing functions (decortication, corm grating, pulverization, and bulla extraction) into a single unit [25]. However, access to these innovations remains uneven, and affordability and adoption rates are still limited in many rural areas.

### 3.11. Research Gaps and Future Directions

Traditional processing methods for bulla remain highly labor‐intensive and variable, whereas scientific evaluation of innovations in processing technology is still emerging. Integrated processing machinery that combines decortication, corm grating, and starch extraction shows promise for improving efficiency and reducing drudgery; however, evidence regarding performance, adoptability among farmers, cost–benefit trade‐offs, and effects on product quality is limited. Despite these promising innovations, the enset value chain, particularly for bulla, lacks standardized processing protocols and quality control systems that could facilitate broader commercialization and market expansion. Without standardized methods, products can vary widely in texture, safety, and nutritional quality, limiting their marketability beyond local informal markets [31].

The absence of recognized quality standards or certification (e.g., hygienic handling guidelines and organic or fair‐trade labeling) also restricts enset product entry into formal retail and export markets, where consistency and traceability are increasingly important [30]. Addressing these gaps through the development of processing guidelines, quality assurance frameworks, and producer training programs could help improve product uniformity and open opportunities for scaling production to meet rising urban and diaspora demand. In addition, there is a lack of research on standardized protocols and quality control frameworks for bulla that could support broader commercialization or entry into formal markets. Studies are needed to develop processing standards, shelf‐life assessments, and safety guidelines, coupled with pilot evaluations of value‐added bulla products (e.g., fortified or convenience foods) that could enhance both nutritional quality and market demand.

### 3.12. Limitations

Limitations of the review include (i) language bias (English only), (ii) potential geographic bias (most studies from Southern Ethiopia), (iii) inconsistent fermentation definitions across primary studies, and (iv) no formal quality assessment of included studies (e.g., using Joanna Briggs Institute or similar tools).

## 4. Conclusion

This review demonstrates that bulla is a nutritionally significant enset food product rich in carbohydrates and important minerals. It is a distinct enset food whose production primarily involves mechanical juice extraction and settling, and does not fundamentally require the microbial fermentation step often attributed to it in the literature. The confusion of considering bulla as fermented food likely stems from concurrent kocho processing or natural changes during its storage. However, there is also fermented bulla with inconsistent literatures on nutritional comparison with bulla. The review also identified bulla as the highest energy density and most commercially valuable food with various reported traditional health benefits, pharmaceutical and biotechnological uses as an excipient in tablet formulations (acting as both a binder and a disintegrant), and deep cultural and economic importance in Ethiopia. However, there are many regional variations in bulla production and processing. There is also a limitation in standardization and quality control, as well as in adopting emerging processing improvements and mechanization efforts to ease labor constraints in traditional processing methods. Therefore, a study is needed to optimize these different processing methods to maximize yield, efficiency, and quality. Such knowledge is crucial to enhance enset cultivation, processing, and marketing that significantly contribute to sustainable food security, income generation, and livelihoods for enset‐dependent communities in Ethiopia. Additionally, further studies are needed on standardized processing protocols, the production of instant bulla for food application, shelf‐life studies, and randomized controlled trials for glycemic index.

## Author Contributions

Getu Weyya: conceptualization, investigation, methodology, and writing original draft; Hirpasa Teressa: conceptualization, writing original draft, and editing; Tariku Tagesse: writing and editing; Habtamu Admassu: supervision, reviewing, and editing; Eneyew Tadesse: supervision, validation, reviewing, and editing.

## Funding

No funding was received for this manuscript.

## Conflicts of Interest

The authors declare no conflicts of interest.

## Data Availability

Data sharing is not applicable to this article as no datasets were generated or analyzed during the current study.
